# The Chimeric Antigen Receptor Detection Toolkit

**DOI:** 10.3389/fimmu.2020.01770

**Published:** 2020-08-11

**Authors:** Yifei Hu, Jun Huang

**Affiliations:** ^1^Pritzker School of Molecular Engineering, University of Chicago, Chicago, IL, United States; ^2^Pritzker School of Medicine, University of Chicago, Chicago, IL, United States

**Keywords:** chimeric antigen receptor (CAR), T cell, detection, cancer immunotherapy, method

## Abstract

Chimeric antigen receptor-T (CAR-T) cell therapy is a promising frontier of immunoengineering and cancer immunotherapy. Methods that detect, quantify, track, and visualize the CAR, have catalyzed the rapid advancement of CAR-T cell therapy from preclinical models to clinical adoption. For instance, CAR-staining/labeling agents have enabled flow cytometry analysis, imaging applications, cell sorting, and high-dimensional clinical profiling. Molecular assays, such as quantitative polymerase chain reaction, integration site analysis, and RNA-sequencing, have characterized CAR transduction, expression, and *in vivo* CAR-T cell expansion kinetics. *In vitro* visualization methods, including confocal and total internal reflection fluorescence microscopy, have captured the molecular details underlying CAR immunological synapse formation, signaling, and cytotoxicity. *In vivo* tracking methods, including two-photon microscopy, bioluminescence imaging, and positron emission tomography scanning, have monitored CAR-T cell biodistribution across blood, tissue, and tumor. Here, we review the plethora of CAR detection methods, which can operate at the genomic, transcriptomic, proteomic, and organismal levels. For each method, we discuss: (1) what it measures; (2) how it works; (3) its scientific and clinical importance; (4) relevant examples of its use; (5) specific protocols for CAR detection; and (6) its strengths and weaknesses. Finally, we consider current scientific and clinical needs in order to provide future perspectives for improved CAR detection.

## Introduction

Chimeric antigen receptor-T (CAR-T) cell therapy is a breakthrough application of adoptive cell therapy (ACT), a novel immunoengineering field where T cells are genetically modified *ex vivo* and infused for anti-tumor, anti-viral, or immunomodulatory effects *in vivo*. At the center of CAR-T cell therapy is the CAR, an engineered immunoreceptor consisting of an extracellular single-chain antibody fragment (scFv) and hinge, a transmembrane region, and intracellular signaling domains. The CAR directs T cells to recognize, activate, proliferate, and kill in response to scFv-driven recognition of tumor-associated antigens (1). Since 2017, two formulations of anti-CD19 CAR-T cell therapy won FDA approval: Kymriah and Yescarta. Both formulations yielded unprecedented 40% complete response rates against relapsed/refractory B-cell leukemia and lymphoma (2, 3). These preliminary successes ignited interest in extending CAR-T cell therapies from hematological malignancies to solid tumors (1). Currently, CARs that target human epidermal growth factor 2 (HER2) and epidermal growth factor receptor variant III (EGFRvIII) against glioblastoma, GD2 disialoganglioside against neuroblastoma, and mesothelin (MSLN) against mesothelioma, are being evaluated in clinical trials (4).

Although CAR-T cell therapy's preliminary clinical success in B cell cancers warrants optimism, there are several challenges in the CAR-T field that need to be addressed: (1) CAR-T cell therapy does not work on solid tumors; (2) clinical non-response/relapse mechanisms in B cell cancers need elucidation; (3) the *in vivo* biology of CAR-T cells in human subjects needs investigation; and (4) the molecular designs of the CAR immunoreceptor need optimization.

Accurate and reproducible CAR detection methods are required to address these challenges. Developing CAR-T cell therapy for solid tumors and elucidating clinical non-response/relapse mechanisms in B cell cancers require methods to stain and sort CAR-T cells from clinical samples for downstream applications, such as multiparameter flow cytometry and next-generation sequencing. Investigating *in vivo* CAR-T cell biology requires methods to track and assess *in vivo* CAR-T cell expansion kinetics, persistence, biodistribution, and effector functions in patients and animal models. Optimizing CAR molecular designs requires methods to characterize CAR signaling and visualize CAR immunological synapse formation at the molecular and cellular levels. Finally, development and application of any CAR detection methods for clinical trial laboratory operations should adhere to Good Clinical Laboratory Practice (GCLP) guidelines, which ensure high data quality, reduced false negative and false positive incidences, and replicability across independent GCLP-compliant laboratories (5).

**Figure 1:**
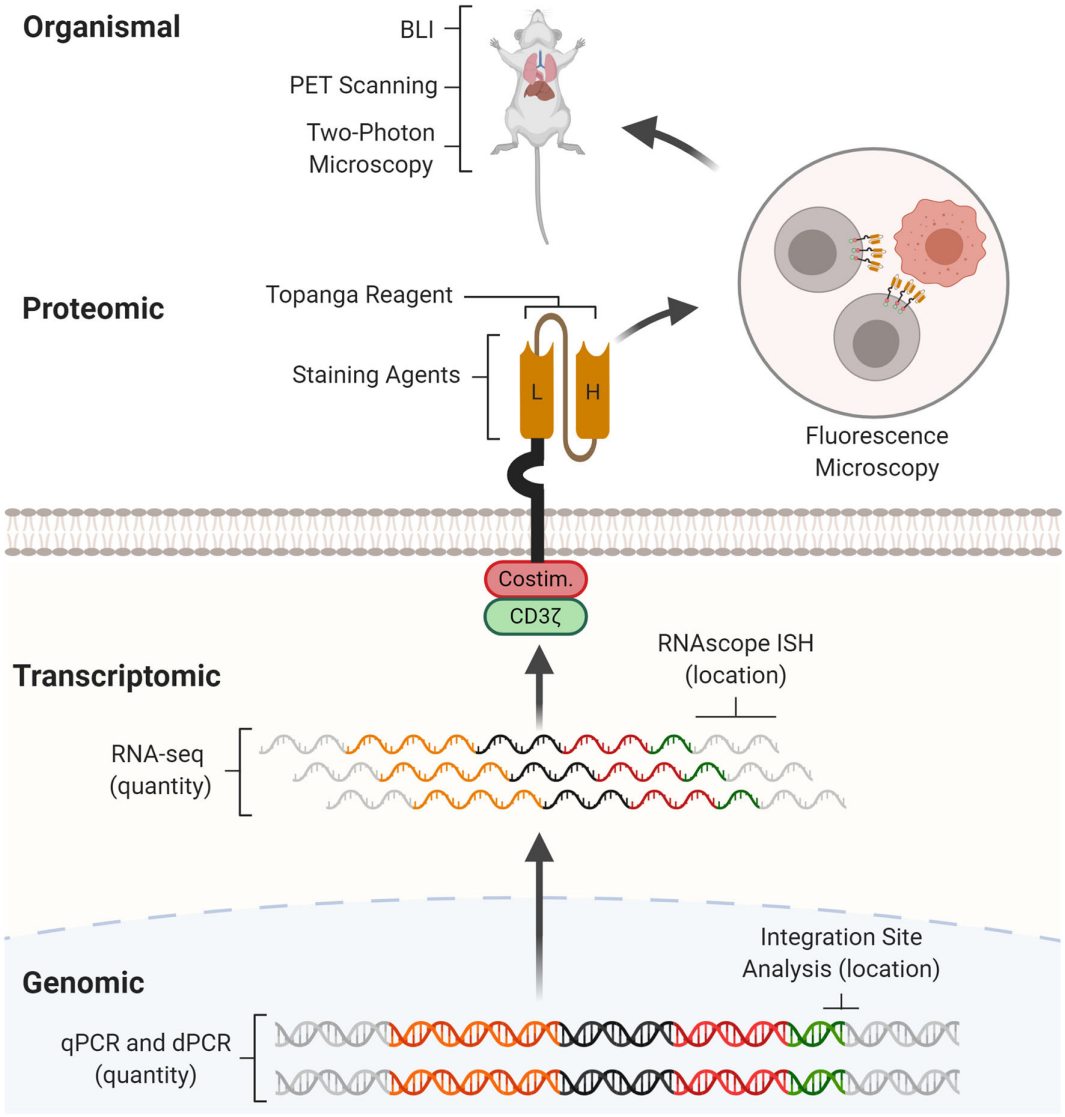
CAR detection methods across multiple levels. CAR detection methods can operate at genomic, transcriptomic, proteomic, and organismal levels. At the genomic level, real-time quantitative PCR (qPCR) and digital PCR (dPCR) measure CAR vector copy number while integration site analysis determines sites of insertional mutagenesis. At the transcriptomic level, RNA-seq measures CAR mRNA abundance while RNAscope *in situ* hybridization (RNAscope ISH) determines the presence and subcellular localization of CAR mRNA molecules. At the proteomic level, staining agents facilitate flow cytometry and western blotting quantification of the CAR protein, while the Topanga reagent detects the CAR via luminescence. The CAR can also be fused with fluorescent proteins for fluorescence microscopy. At the organismal level, bioluminescence imaging (BLI) and positron emission tomography (PET) scanning determines the distribution of CAR-T cells between organs, while two-photon microscopy tracks single CAR-T cells in tissue.

Here, we review current CAR detection methods. After describing the target and importance of each CAR detection method, we will discuss experimental protocols, examples of its application, as well as its strengths and weaknesses. Wherever possible, we will provide perspectives for method improvements. We will introduce CAR detection methods in the order of the level at which they operate: genomic, transcriptomic, proteomic, and organismal (Figure 1). By facilitating experimental design and planning, this review aims to catalyze basic, immunoengineering, and clinical research.

## CAR Detection at the Genomic Level

**Figure 2:**
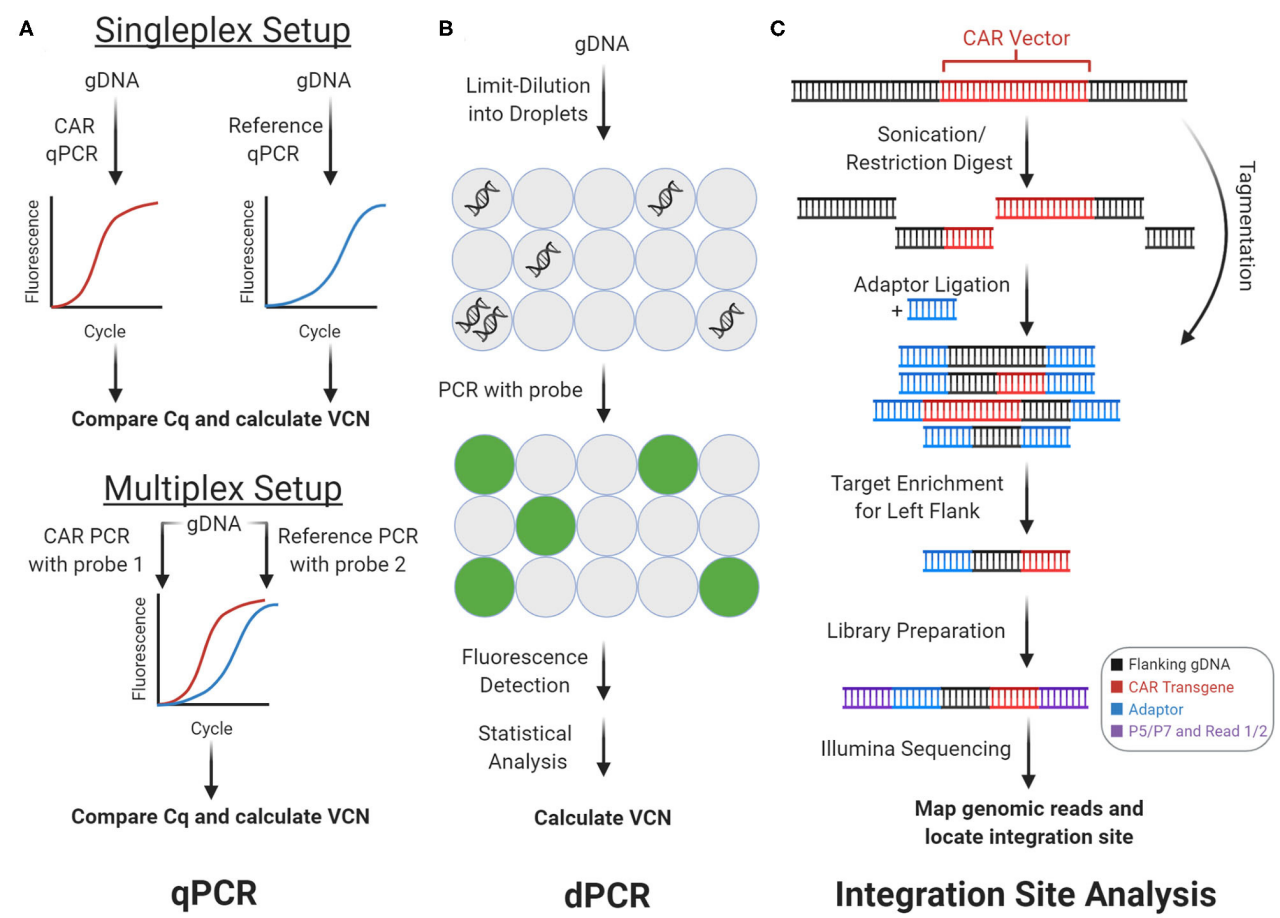
Genomic CAR detection. Real-time quantitative PCR (qPCR) and digital PCR (dPCR) measure CAR vector copy number (VCN) while integration site analysis determines sites of insertional mutagenesis. **(A)** With qPCR, target amplicons are amplified from genomic DNA (gDNA) with fluorescent probes. C_q_ is calculated from fluorescence tracked over PCR cycles, which measures copy number. In the singleplex setup, vector and reference gene are amplified separately. In the multiplex setup vector and reference are amplified concurrently using two independent probes. **(B)** With dPCR, gDNA is partitioned into tiny droplets. Most droplets contain zero or one template copies. Target amplicons are amplified from each droplet separately, and the proportion of fluorescent droplets measures copy number. **(C)** With integration site analysis, gDNA is fragmented and ligated with adaptors in two steps with sonication or restriction enzymes, or in one step with tagmentation. Fragments containing either of the CAR vector flanks (left flank shown here) can be enriched and prepped for sequencing with multiple rounds of PCR. Mapping the reads to the genome determines sites of insertional mutagenesis.

During the CAR manufacturing process, T cells are virally transduced with a CAR vector, which semi-randomly integrates into the T cell's genome. There are three main methods for detecting the integrated CAR vector: real-time quantitative polymerase chain reaction (qPCR, Figure 2A), digital polymerase chain reaction (dPCR, Figure 2B), and integration site analysis (Figure 2C). Using genomic DNA (gDNA), qPCR and dPCR determine the frequency while integration site analysis determines the genomic location of the CAR vector. Since gDNA is more stable than mRNA, proteins, or cryopreserved biospecimens, experiments involving these methods can be easier to coordinate. Importantly, these methods can help evaluate and optimize the safety profiles of alternative non-viral techniques for CAR vector delivery, including CRISPR/Cas9 and transposon-mediated insertion (6, 7).

### Real-Time Quantitative Polymerase Chain Reaction

qPCR measures the frequency of integrated CAR vector in the genome (Figure 2A). Using target-specific primers and fluorescent probes, qPCR amplifies and quantifies an amplicon over PCR cycles. The quantitation cycle (C_q_), when fluorescence exceeds background levels, measures an amplicon's relative (compared to another amplicon) or absolute (compared to a standard curve) copy number. With CAR-T cells' gDNA and CAR-specific primers and probes, qPCR can measure vector copy number (VCN)—the average vector copies per genome. VCN estimates CAR vector delivery efficiency and CAR-T cells' representation in a cell pool. Hence, VCN is an important quality metric for clinical-grade CAR-T cell infusions and a technical benchmark that non-viral forms of CAR vector delivery, such as transposon-mediated delivery, must improve upon (7). In both CAR-T cell research and clinical settings, qPCR helps monitor VCN from patient blood gDNA over the course of CAR-T treatment. These results have consistently shown strong correlations between CAR-T cell expansion, persistence, clinical response, and grade of side effects across multiple types of B cell cancers (2, 3, 8).

Optimized CAR qPCR protocols have been developed to detect the anti-CD19 (clone FMC63) scFv. Wang et al. (9) developed and validated TaqMan qPCR primers and probes for a ~130 bp amplicon from the FMC63 nucleotide sequence. In their assay, qPCR was performed side-by-side against FMC63 and *GAPDH* to measure CAR copy number and genome copies, respectively. These two measured values were used to calculate VCN. In addition to robustness across replicates, their qPCR assay achieved a minimum detection limit of 10 CAR copies per μL of blood and linear signal between 10 and 10^7^ copies/μL. However, a singleplex design increases sample and reagent use, decreases throughput, and introduces pipetting noise. Multiplexed qPCR can address these issues. Kunz et al. developed a single copy gene-based duplex qPCR assay (SCG-DP-PCR) to measure VCN. In their assay, the FMC63 scFv and RNaseP (*RPPH1*) were simultaneously qPCR-amplified from the same gDNA sample using two independent fluorescent probes. Using *RPPH1* as an internal control, their duplex setup resulted in similar efficiency as the corresponding singleplex setup. As proof-of-principle, they used SCG-DP-PCR to measure longitudinal VCN from three sets of CAR-T patient blood gDNA samples (10).

Overall, qPCR is a common and robust assay for monitoring CAR VCN. With well-designed primers and probes, qPCR is rapid, easily performed, and trustworthy for clinical use. qPCR can measure CAR vector delivery efficiency, on-treatment expansion kinetics, and persistence to predict clinical response (2, 3, 8). As such, qPCR machines optimized and certified for CAR VCN measurements are now available commercially. However, qPCR has notable weaknesses. While robust at the population level, qPCR cannot differentiate subtle copy number differences. Hence, qPCR for VCN at the single-cell level is expected to be noisy and has never been implemented (11). Other than VCN, qPCR cannot determine the CAR-T cells' phenotype or whether the CAR is actually expressed. CAR expression depends on the chosen viral promoter, local chromatin architecture, regulatory elements (i.e., promoters, enhancers, insulator sequences), DNA methylation, and biological noise. Hence, qPCR overestimates the number of functional CAR-T cells in a given population. Furthermore, the reliability of qPCR results depends on the target specificity of the primers and probes. In conclusion, qPCR is a clinically useful assay for monitoring VCN, but RNA-seq and flow cytometry may be more useful methods for determining CAR-T cell functionality in research settings.

### Digital Polymerase Chain Reaction

Like qPCR, dPCR also measures VCN (Figure 2B). However, dPCR measurements are more sensitive and precise. In brief, the gDNA template is partitioned into tiny droplets. Most droplets contain zero or one template molecule. PCR amplification of the target amplicon with a fluorescent probe occurs separately within each droplet. Subsequently, the droplets are flowed through an excitation source and detector, which measures each droplet's fluorescence. With Poisson statistics, the proportion of fluorescent droplets is used to calculate copy number (12). While qPCR relies on continuous intermediate fluorescence measurements from PCR cycles, dPCR relies only on end-point fluorescence. Therefore, dPCR is robust to amplification kinetics and suppresses noise. Like with qPCR, dPCR can be multiplexed to decrease sample and reagent use, increase throughput, and decrease pipetting noise.

Fehse et al. developed a duplex dPCR assay to concomitantly probe the anti-CD19 CAR and a reference gene. Their duplex dPCR assay achieved a minimum detection limit of 0.01% CAR-transduced cells from 100 ng of gDNA. As proof-of-principle, they applied their assay on five sets of Yescarta patient blood gDNA samples (13). The enhanced sensitivity from dPCR also enables single-cell VCN measurements. Santeramo et al. recently developed dPCR to measure VCN in single lentivirally transduced T cells. In their assay, target amplicons were first pre-amplified to generate sufficient template material, prior to dPCR for vector and reference amplicons. Their single-cell assay generated results that were consistent with bulk measurements (14). However, their method has yet to be applied for CAR VCN measurements.

As a more sensitive assay for monitoring VCN, dPCR shares strengths and weaknesses with qPCR. Unlike qPCR, the increased sensitivity allows dPCR to measure VCN in single cells. Single-cell VCN measurements can capture cell-cell heterogeneity in transduction efficiency within a CAR-T cell infusion, which may impact clinical efficacy. However, dPCR-compatible machines are rarer, and dPCR reactions are more costly.

### Integration Site Analysis

The presence and genomic location of the integrated CAR vector can be assayed via integration site analysis (Figure 2C). During CAR transduction, the CAR vector is randomly inserted into the genome (i.e., insertional mutagenesis), which can disrupt genes, trigger premalignant T cell proliferation, promote CAR-T cell efficacy, and influence clinical response (15–17). Integration site analysis maps sites of insertional mutagenesis using next-generation sequencing. Importantly, integration site analysis can characterize the integration loci biases of different CAR vector delivery techniques. In brief, integration site analysis involves fragmentation, PCR, and analysis steps. First, gDNA from the CAR-T cell sample is fragmented via restriction enzymes, transposases, or sonication. If necessary, adaptors are ligated onto the resulting DNA fragments. Then, fragments containing the CAR vector and flanking genome are enriched by PCR amplification, using a primer that anneals on the adaptor paired with a primer that anneals on the CAR vector. When this amplicon is sequenced, reads begin in the vector and extend into flanking human DNA, which can be aligned onto the human genome to reveal the integration loci.

Historically, integration site analysis employed restriction enzymes to fragment gDNA (18). However, restriction enzymes generated inconsistent results that depended on which restriction enzyme was used (19). The more up-to-date integration site pipeline for paired-end reads (INSPIIRED) eliminates restriction enzyme bias by using sonication for fragmentation. INSPIIRED includes well-documented steps for sonication, library preparation, Illumina paired-end sequencing, and bioinformatic site-calling (20, 21). INSPIIRED has been employed on anti-CD19 CAR-T cell therapy infusion samples, which showed that insertional mutagenesis near genes in cell-signaling and chromatin modification pathways predicted clinical response (15). The final and more elegant method for integration site mapping uses transposases to combine the fragmentation and ligation steps (i.e., tagmentation). In one step, the transposase agnostically fragments gDNA and inserts an adaptor for PCR amplification and Illumina sequencing. The tagmented gDNA can simultaneously be used for integration site analysis and chromatin accessibility profiling (via ATAC-seq) via the recently developed vector integration analysis with epigenomic assay (EpiVIA), which can be applied at both the bulk and single-cell level (22). In a clinical case study, Mu transposase-enabled integration site analysis characterized how lentiviral insertion of the CAR vector disrupted *TET2* and led to massive (94% of blood CD8^+^ T cells) CAR-T cell expansion (16, 19). In addition, Tn5 transposase-enabled integration site analysis was employed to compare integration sites between γ-retrovirus, lentivirus, and *piggyBac* transposon-mediated gene transfer. Compared to viral transduction, the *piggyBac* transposon integrated less often near transcriptional start sites and more often into genomic safe harbors (7).

Overall, integration site analysis is a clinically relevant CAR detection method for locating sites of insertional mutagenesis. Newer integration site analysis pipelines involving transposases significantly streamline benchwork and enable EpiVIA, which combines integration site analysis and ATAC-seq at the bulk and single-cell levels (22). Furthermore, the abundance of each CAR-T cell clone (each of which can be assumed to harbor unique integration sites) can be bioinformatically inferred from integration site sequencing data (23). However, capture efficiencies for most CAR integration site analysis methods are unavailable. Where available, capture efficiencies are notably poor. At least three Mu transposase-enabled integration site analysis replicates were required to capture all six integration sites in a cell line (19). Since polyclonal CAR-T cell populations contain far more than six integration sites, integration site analysis is unlikely to capture all integration sites, especially those from rare clones. With single-cell EpiVIA, only ~200 integration sites were detected from 700 M read pairs in ~5,000 CAR-transduced T cells, which was notably far from saturation (22). Hence, capture efficiencies should be better characterized, and protocol improvements are needed to assay rarer clones.

## CAR Detection at the Transcriptomic Level

**Figure 3:**
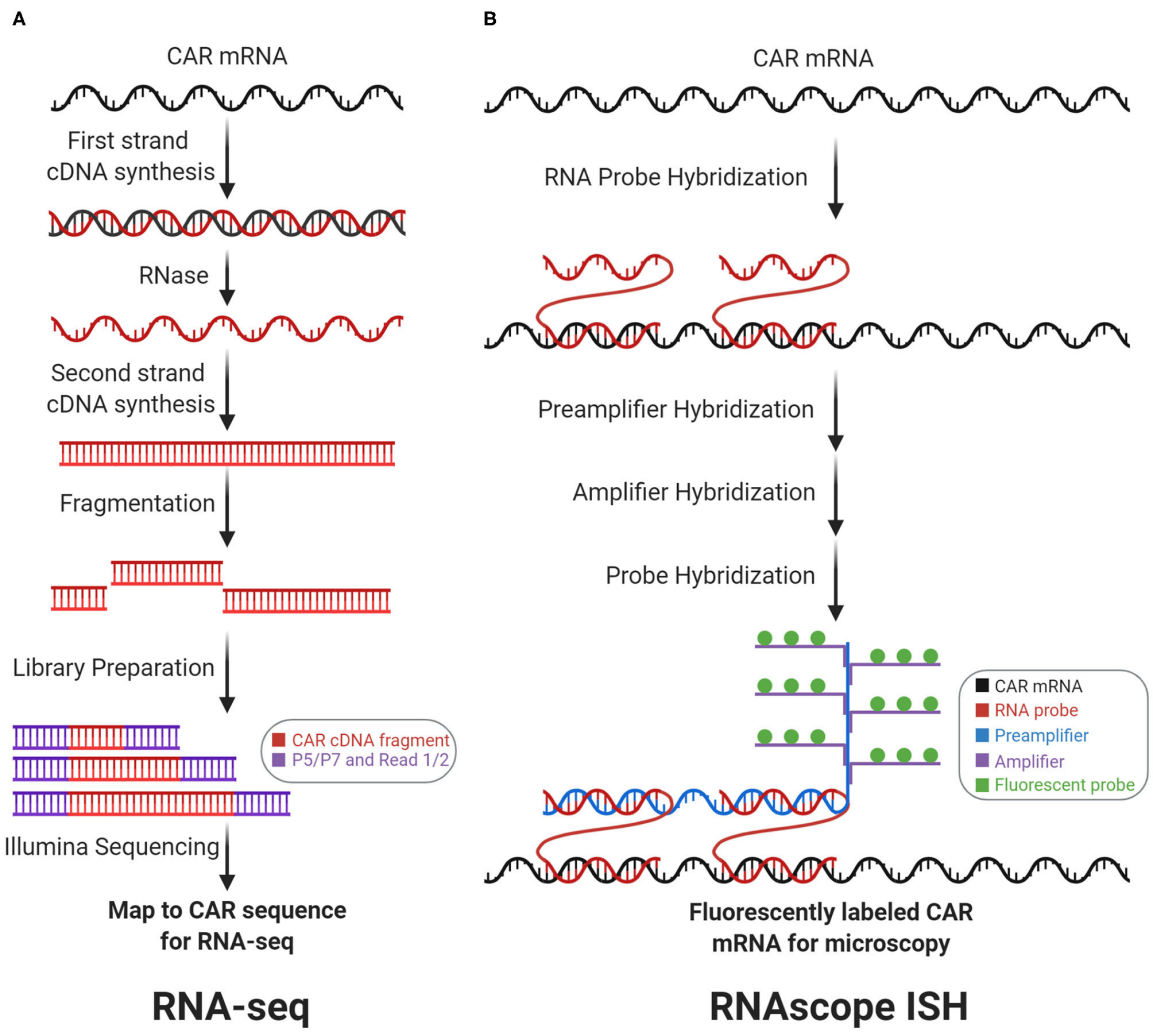
Transcriptomic CAR detection. RNA-sequencing (RNA-seq) and RNAscope *in situ* hybridization (RNAscope ISH) measure CAR mRNA abundance and subcellular localization, respectively. **(A)** With RNA-seq, CAR mRNA is first converted to cDNA, which is then fragmented and prepped for sequencing. Counting the number of reads that map to the CAR sequence measures CAR mRNA abundance. **(B)** With RNAscope ISH, the CAR mRNA is first hybridized with target-specific RNA probes. Subsequently, this complex is hybridized with the preamplifier, amplifier, and fluorescent probes to form a fluorescently labeled CAR mRNA complex for fluorescence microscopy.

After integration into the genome, the CAR vector is transcribed into mRNA. There are two main methods for detecting CAR mRNA: RNA-sequencing (Figure 3A) and RNAscope *in situ* hybridization (Figure 3B). These methods determine the abundance and subcellular location of the CAR mRNA, respectively. Detection of CAR mRNA can be more functionally relevant than detection of the CAR vector, since CAR mRNA is closer to CAR protein, which exerts biological functions.

### RNA-Sequencing

CAR mRNA abundance can be quantified by RNA-sequencing (RNA-seq, Figure 3A). CAR mRNA quantity depends upon genomic factors: VCN, viral promoter strength, local chromatin architecture, regulatory elements, and DNA methylation. Importantly, CAR mRNA quantity drives antigen-independent tonic signaling (24) and amount of translated CARs on the cell surface influences antigen-sensitivity, NFAT signaling, and cytokine production (25). In general, RNA-seq quantifies mRNA by converting mRNA to cDNA via reverse transcription. The cDNA can subsequently be fragmented, sequenced, and aligned to gene sequences to measure mRNA abundance. RNA-seq can correlate CAR mRNA abundance with transcriptional profiles. For instance, Zhang et al. utilized RNA-seq on anti-CD19 CAR-T patient samples to measure correlation between CAR and *CD19* expression (26). These analyses also apply at the single-cell level. Sheih et al. utilized single-cell RNA-seq to quantify CAR mRNAs and interrogate transcriptional profiles in CD8^+^ T cells from CAR-T infusion products. CAR mRNA quantification helped to distinguish between CAR-transduced vs. non-transduced cells in their single-cell dataset (27). Prospectively, RNA-seq may also help characterize how viral promoters influence CAR transcription and *in vivo* differentiation. For example, with anti-CD19 CAR-T cell therapy, Kymriah employs the elongation factor 1-α (EF-1α) promoter while Yescarta employs the murine stem cell virus (MSCV) promoter. Although the EF-1α promoter drives higher and more consistent murine *in vivo* transcription than the MSCV promoter (28), no studies have determined whether this difference influences CAR-T cell functionality, differentiation, or clinical outcomes.

RNA-seq is now a routinely employed biological assay with many published protocols, commercial kits, and analysis pipelines. By integrating the genomic factors that influence CAR expression into a single readout, RNA-seq for the CAR mRNA arguably provides more clinically and biologically relevant data than qPCR for the CAR vector. Furthermore, in single-cell RNA-seq datasets, CAR mRNA quantification can help differentiate CAR-T cells from non-CAR-T cells during analysis (27). However, mRNA-seq notably cannot capture factors that influence CAR translation, such as ribosome, initiation factors, and amino acid availability. Therefore, flow cytometry or western blotting for the CAR protein may be superior methods for determining CAR functionality.

### RNAscope *in situ* Hybridization

Both the quantity and subcellular localization of the CAR mRNA can be determined by RNAscope *in situ* hybridization (RNAscope ISH, Figure 3B). RNAscope ISH utilizes RNA-specific oligonucleotide probes that anneal with a targeted RNA molecule in fixed and permeabilized cells, to generate fluorescence signals for microscopy. Dual target probes (for specificity) and additional adaptor probes (for signal amplification) enable detection, localization, and visualization at the single-molecule level. Using orthogonal sets of probes, RNAscope ISH can be multiplexed—CAR mRNA can be simultaneously detected along with other target mRNA on the same slide (29). Furthermore, RNAscope ISH can correlate a CAR-T cell's CAR mRNA quantity with the CAR-T cell's relative location within a tissue sample. For instance, RNAscope ISH (to detect the CAR mRNA's 3′-untranslated region) was employed to visualize anti-EGFRvIII CAR-T cell infiltration into glioblastoma tumor sections before and after intravenous infusion. This study showed active trafficking of anti-EGFRvIII CAR-T cells into tumor regions, which correlated with EGFRvIII downmodulation on tumor cells (30). Furthermore, RNAscope ISH was employed to show anti-ROR1 and anti-BCMA CAR-T cell biodistribution and tissue trafficking in xenograft tumor models (31).

RNAscope ISH is a specialized tool with many strengths: (1) spatial resolution spanning the single-molecule and cellular levels; (2) ability to probe subcellular mRNA localization; (3) capability for multiplex detection; and (4) compatibility with microscopy. However, RNAscope ISH requires fixed and permeabilized cells, hence it cannot be used for live-cell RNA imaging. Studies have yet to take advantage of this method's unique strengths. For example, current studies use RNAscope ISH to distinguish CAR-T cells from non-CAR-T cells in tissue sections, with only limited use of its multiplexing capabilities. Furthermore, no studies have yet analyzed how CAR mRNA subcellular localization (e.g., near cell membrane) may influence CAR mRNA stability, degradation, or translation.

## CAR detection at the Proteomic Level

**Figure 4:**
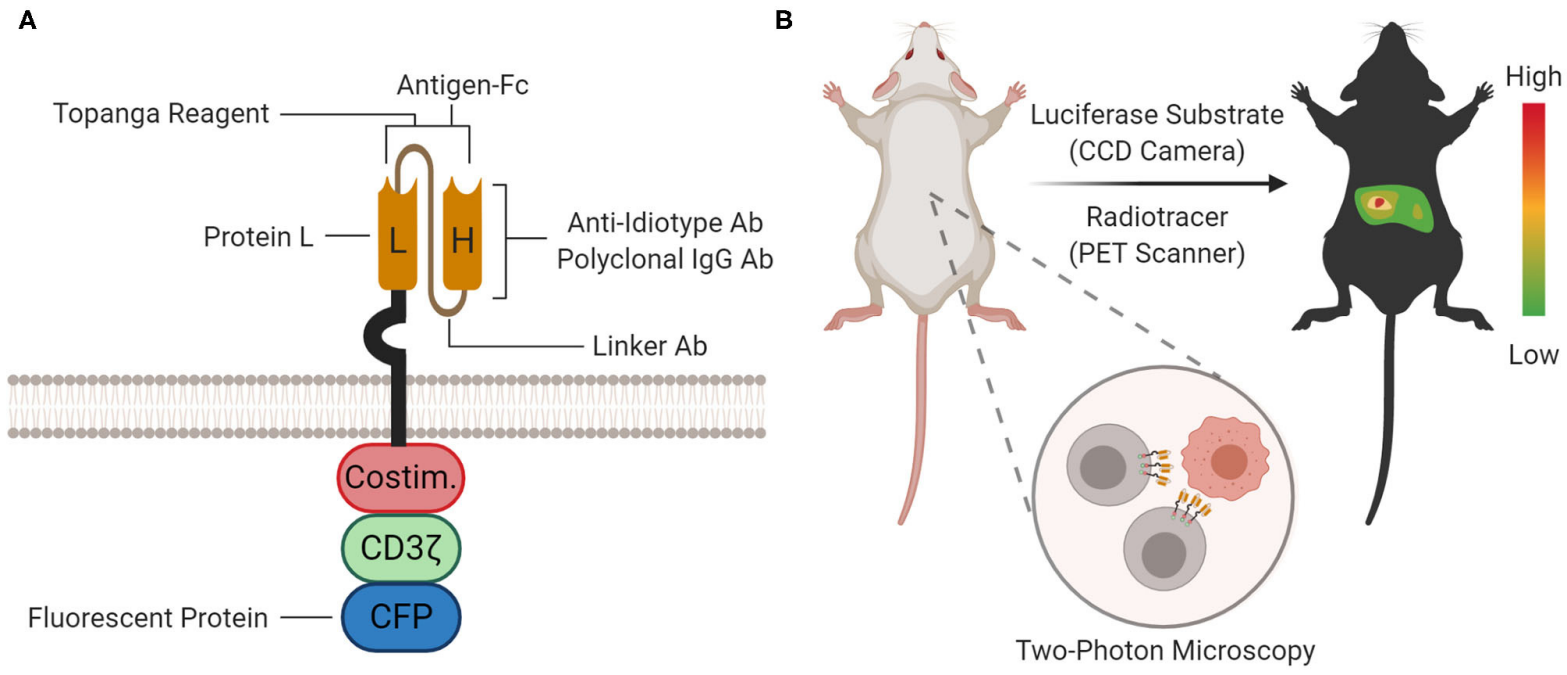
Proteomic and Organismal CAR Detection. **(A)** At the proteomic level, the CAR protein can be detected with staining agents (for flow cytometry and immunoprecipitation), Topanga reagent (for luminescence), or fused fluorescent proteins [for microscopy and flow cytometry; cyan fluorescent protein (CFP) is shown as an example]. The approximate location or binding site for each method is depicted on the cartoon. **(B)** At the organismal level, the biodistribution of CAR-T cells between organ compartments can be measured by bioluminescence imaging (BLI) or positron emission tomography (PET) scanning using a luciferase substrate or radiotracer, respectively. Furthermore, single CAR-T cells can be tracked in tissue with two-photon microscopy.

After translation from CAR mRNA, the CAR protein drives antigen-dependent signaling. Unlike detection at the genomic or transcriptomic levels, detection at the proteomic level can directly evaluate CAR functionality. The CAR protein can be detected by flow cytometry (with staining agents), luminescence (with Topanga reagent), immunoprecipitation (with staining agents), and microscopy (with fluorescent protein fusions). Figure 4A depicts where each detection reagent acts.

### Staining Agents for Flow Cytometry

The presence and quantity of the CAR protein on the cell surface can be assayed via fluorescent CAR-staining agents and flow cytometry. In addition to CAR protein quantitation, these staining agents also enable multicolor flow cytometry-based profiling and fluorescence-activated cell sorting. CAR-staining agents were instrumental in illuminating factors that impact CAR-T cell clinical efficacy, including T cell subset composition, CAR downmodulation after antigen engagement (25, 32), and CAR-T cell trogocytosis (33). Although many CAR-staining agents exist, a comparison of sensitivity and specificity metrics between these staining agents has yet to be performed. Each staining agent's target site and general properties are summarized in Figure 4A and Table 1, respectively.

**Table 1:** CAR staining reagents.

**Property**	**Anti-IgG antibodies**	**Protein L**	**Antigen-Fc**	**Anti-idiotype antibody**	**Anti-linker antibody**
One-step staining	Yes	No	No	Yes	Yes
Compatibility w/antibody panels	Inconsistent	No	Yes	Yes	Yes
Compatibility w/FcX reagents	Inconsistent	No	Some	Yes	Yes
Reagent stability	High	High	Often low	High	High
Specificity for CAR	Low	Low	High	High	High
Access to academic labs	Easy	Easy	Easy	Hard	Hard

Two CAR-staining agents target IgG-like fragments: polyclonal anti-IgG antibodies and Protein L. Polyclonal anti-IgG (often of goat origin) are commonly used as secondary antibodies to stain IgG-like fragments. As polyclonal reagents, they have significant batch-to-batch variation. Although they are provided by a variety of vendors, the polyclonal goat anti-mouse F(ab)_2_ from Jackson ImmunoResearch Laboratories is the most widely used, and was historically used to characterize the anti-CD19 CAR in Yescarta (34). Protein L is a *Peptostreptococcus magnus* bacterial surface protein that binds to many immunoglobulin kappa (κ) light chains, including human V_K_I, V_K_III, V_K_IV, and murine V_K_I, without interfering with the immunoglobulin's antigen-binding site. In addition to whole antibodies, Protein L can also bind light chains on scFv (35). Zheng et al. optimized Protein L as a CAR-staining reagent for flow cytometric detection: biotinylated Protein L (1 μg per million lymphocytes in 200 μL) is applied followed by fluorophore-labeled streptavidin. Successfully staining was achieved with a variety of CARs, including CARs containing human scFv (anti-EGFRvIII, anti-VEGFR2), murine scFv (anti-CD19, anti-CSPG4), and humanized scFv (anti-HER2, anti-PSCA). However, their method requires multiple cell washes before Protein L staining, since carry-over immunoglobulin in serum or culture media must be strictly removed. Furthermore, their results suggest their method may have worse stain index compared to polyclonal anti-IgG antibodies (36). Protein L has been used to characterize masked CARs with tumor-specific activation (37), CAR downmodulation (25), tonic signaling (38), and to activate CAR-T cells via Protein L that is bound on plates (25).

Although both polyclonal anti-IgG antibodies and Protein L are relatively cheap CAR-staining reagents, they share significant shortcomings: (1) cross-reactivity with non-CAR IgG-like proteins on the cell surface, requiring stringent washing before and after staining; (2) incompatibility with antibodies and many FcX blocking reagents during multicolor flow cytometry, requiring multiple staining and washing steps; (3) cannot independently stain different CARs on a dual-CAR expressing T cell; and (4) cannot stain CARs with synthetic scFv.

Antigen-Fc is a CAR-staining agent that takes advantage of the CAR's binding affinity for its target antigen. Antigen-Fc are chimeric proteins with an N-terminal target antigen fused to a C-terminal Fc fragment (often from human IgG1). Due to the Fc fragment, antigen-Fc dimerizes under non-reducing conditions in solution and can be purified via Protein A beads. To stain CAR-T cells, antigen-Fc is applied, followed by a secondary staining step with fluorophore-labeled anti-Fc or anti-biotin/streptavidin (if the antigen-Fc was biotinylated). Alternatively, the antigen-Fc is directly conjugated with a fluorescent dye, which eliminates the secondary staining step. Biochemically, antigen-Fc binds with antibody-like specificity and affinity. Antigen-Fc has been used to evaluate novel CAR constructs (39), to independently measure expression of each CAR in dual CAR-T cells (40), and to activate CAR-T cells via antigen-Fc that is bound on plates (39). Antigen-Fc, including CD19-Fc, HER2-Fc, and PSCA-Fc, are commercially available from many vendors.

CD19-Fc is of special interest, due to the success of anti-CD19 CAR-T cell therapy and availability of patient samples for research. De Oliveira et al. expressed CD19-Fc for CAR-T cell staining and found that exon 4 of the CD19 ectodomain (CD19ecto) is required for binding to the anti-CD19 (FMC63) CAR used in Yescarta and Kymriah. However, their staining results show inferior stain index than either polyclonal anti-IgG antibodies or Protein L, hinting at issues with protein quality (41). Indeed, CD19ecto aggregates in higher-order disulfide-bonded oligomers and is notorious for being a difficult-to-express protein (42). The crystal structure of CD19ecto bound to a B43-Fab shows that CD19ecto can form a unique elongated β-sandwich, which may be difficult to fold properly within overexpression systems (43). In response to technical challenges with CD19ecto production, Lobner et al. expressed a novel chimera consisting of an N-terminal CD19ecto with a C-terminal human serum albumin domain 2 (AD2). Compared to CD19ecto, the CD19-AD2 chimera is easier to produce, monomeric, and effectively binds and stains the anti-CD19 (FMC63) CAR (44).

In conclusion, although antigen-Fc are more CAR-specific than polyclonal anti-IgG antibodies and Protein L, antigen-Fc also have notable disadvantages: (1) more expensive; (2) possible decreased stability in solution compared to traditional antibodies; (3) may be incompatible with FcX blocking reagents; and (4) the Fc fragment may non-specifically bind Fc receptors. Future iterations of antigen-Fc may involve engineered Fc regions that enable compatibility with FcX blocking reagents and disable non-specific binding of Fc receptors. Furthermore, future iterations of antigen-Fc may also take advantage of higher valency binding. Antigen-Fc are analogous to MHC-multimers, which stain T cells via TCR-binding (45, 46). Research on MHC-multimers have shown that higher valency staining reagents improve sensitivity via higher avidity binding. For example, MHC-dodecamers (12-valency) and MHC-dextramers (>>4-valency) are more sensitive than MHC-tetramers (4-valency) (47, 48). However, CAR-staining reagents with higher target valency than antigen-Fc (2-valency) have yet to be constructed. The potential sensitivity enhancement from higher avidity binding has yet to be determined. Higher sensitivity antigen-Fc variants may facilitate staining and analysis of CAR-T cells with low CAR expression due to genome position effects on the CAR vector or CAR downmodulation.

Another CAR-staining agent is anti-idiotype antibodies. Anti-idiotype antibodies specifically bind the variable regions of a particular scFv. Furthermore, anti-idiotype antibodies enable immunohistochemical staining and can potentially block CAR ligation in an *in vivo* setting. Jena et al. developed and characterized a novel monoclonal anti-idiotype antibody (clone 136.20.1) against the anti-CD19 (FMC63) scFv from immunized mice. Their results show 136.20.1 has a lower detection limit of 0.1%, is compatible with microscopy and immunohistochemistry, and inhibits the effector functions of anti-CD19 CAR-T cells (49). This antibody has since been used to characterize CAR-T cells in preclinical studies (16, 24) and clinical trials (50). Interestingly, 136.20.1 was also used to create a novel anti-idiotype CAR as a cellular antidote and kill switch during therapy (51). Other anti-idiotype antibodies include clone 1A7, which can detect the anti-GD2 (14g2a) CAR (52).

Notable advantages of anti-idiotype antibodies include high reagent stability, low background staining, compatibility with antibody panels in multicolor flow cytometry, and the ability to discriminate between different types of CARs. However, most anti-idiotype antibodies are difficult-to-obtain and commercially unavailable.

Finally, Kite Pharma developed rabbit monoclonal antibodies against two commonly used linkers in the CAR scFv: clone KIP-1 against the Whitlow linker and clone KIP-4 against the G_4_S linker. These linkers connect the heavy and light chains in the scFv. KIP-1 can detect (via flow cytometry) and activate CAR-T cells with the Whitlow linker (i.e., Kymriah and Yescarta) (53). KIP-1 was subsequently used in Kite Pharma and Gilead-sponsored studies (54). However, these linker antibodies are unlikely to be accessible to most academic labs without industry sponsorship.

### Biochemical Assays

The CAR's presence and binding competence can be assayed by luminescence using the Topanga reagent. Gopalakrishnan et al. developed the Topanga reagent, a chimeric protein consisting of the N-terminal CAR-antigen fused to a C-terminal marine luciferase, NLuc. Incubation of the Topanga reagent with CAR-T cell mixtures facilitated luminescent detection. The Topanga reagent's exceptional sensitivity allows it to detect CAR-binding in a mixture of 0.001% CAR-T cells out of 1 million PBMCs (55). Although the Topanga reagent cannot determine percentage of CAR-expressing cells, it might be useful for quality control during manufacturing and testing the binding functionality of the CAR.

CAR signaling can be assayed by immunoprecipitation (IP) or co-immunoprecipitation (co-IP) using Protein L-conjugated beads for analysis of CAR post-translational modifications and interaction partners (36). Protein L-conjugated beads bind to the light chain of the CAR scFv, and can pull down CAR interaction partners for western blotting or mass spectrometry. Ramello et al. utilized this approach to pull down CAR immune complexes. Complexes were analyzed by tandem liquid chromatography-mass spectrometry, to identify 253 CAR interaction partners enriched within 15 canonical pathways. Notably, their analysis demonstrated that 2nd-generation CARs associate with a constitutively phosphorylated CD3ζ, which correlates with stronger phosphorylation of downstream signaling proteins (56). In addition to Protein L beads, CAR IP can be performed on epitope-tagged CAR receptors. Salter et al. used the 9-amino acid Strep-tag II to tag the CAR between the scFv and the hinge. Their co-IP analysis showed that endogenous Lck and CD28 differentially associate with CD28-based and 4-1BB-based CARs in the absence of signaling (57). IP with epitope tags is expected to be more target-specific than IP with Protein L, since Protein L is known to interact with IgG-like molecules. However, these epitope tags must not interfere with CAR function. Furthermore, current FDA-approved anti-CD19 CAR designs do not have a convenient epitope tag for IP.

### Microscopy

CAR trafficking and immunological synapse (IS) formation can be visualized by microscopy. Importantly, confocal and total internal reflection fluorescence (TIRF) microscopy can probe CAR signaling and inform CAR engineering.

Confocal microscopy visualizes the CAR with high spatial resolution. Effector and target cells can be adhered to glass slides, allowed to interact, and fixed prior to imaging. Using this approach, Davenport et al. found that CAR-T cells form non-classical IS with multifocal Lck microclusters that may facilitate serial killing (58). In addition, Long et al. utilized confocal microscopy with Cerulean-tagged CAR to show aggregation of the anti-GD2 CAR on the cell surface, which may contribute to antigen-independent tonic signaling (38). However, this setup limits spatial resolution because the CAR IS lies on a vertical imaging plane formed through horizontal cell-cell interactions (59). Xiong et al. circumvented this limitation using confocal microscopy with a vertical cell-pairing system, which flips the CAR IS onto a horizontal imaging plane. Their setup revealed that characteristics of the IS, including antigen clustering, lytic granule polarization, and distribution of key signaling molecules, predict CAR-T cell efficacy *in vivo* (60).

In live cells, the CAR can be visualized at the molecular level via lipid bilayer experiments in conjunction with TIRF microscopy. TIRF microscopy excites fluorophores by inducing an evanescent field near the interface between two media with different refractive indices. In this setup, the CAR-T cell interacts with antigen on glass-supported lipid bilayers to form an IS on the horizontal plane. The evanescent field selectively excites fluorophores near this plane. CAR proteins at this interface can be directly or indirectly detected, via fluorescently labeled CAR or CAR antigen (61). Using TIRF, Xiaolei et al. characterized recruitment of CAR microclusters to the CAR IS, and found that the CAR IS disassembles quicker than the classical TCR IS (62).

With either TIRF or confocal microscopy, fluorescently labeling the CAR for direct detection is preferred over labeling the CAR antigen. Labeling the CAR allows CAR tracking outside of the IS and in resting CAR-T cells. One common method is to chimerically tag fluorescent proteins, such as green fluorescence protein derivatives, to the CAR C-terminus. In addition to enabling direct CAR visualization, this method also facilitates CAR quantification via flow cytometry. Walker et al. utilized cyan fluorescence protein-labeled CAR to measure anti-CD19 CAR downmodulation after antigen engagement. However, this tagging was not possible with all CAR constructs. They reported that an anti-ALK CAR tagged with cyan fluorescence protein failed to express (25). Similarly, Morrissey et al. engineered enhanced green fluorescence protein-labeled CARs for phagocytosis that direct macrophages to engulf target cells. Trafficking of these CARs was studied via live-cell imaging (63). For CARs that are not amenable to fluorescent protein fusion, an alternative method is staining the extracellular regions of the CAR with fluorescent Fabs or scFvs immediately before microscopy. These Fabs should neither block the CAR antigen binding site nor influence CAR trafficking or mechanotransduction. Sasmal et al. utilized this method to label the TCR with an anti-TCRβ scFv for FRET studies (64). However, this method has yet to be applied onto CARs.

Finally, CARs have yet to be visualized with super-resolution microscopy or lattice light-sheet microscopy (LLSM). These emerging technologies can significantly improve spatial and temporal resolution. Rosenberg et al. utilized LLSM to visualize TCR dynamics, which were correlated with T cell signaling states (65, 66). However, similar experiments to characterize CAR dynamics have not yet been performed. Nerreter et al. utilized stochastic optical reconstruction microscopy, a form of super-resolution microscopy, to image CD19 expression on multiple myeloma patient cancer cells, which was compared with flow cytometry data. Their analysis established a sensitivity threshold for CAR-T cell efficacy (67). However, similar studies to visualize the CAR itself have not been conducted.

## CAR Detection at the Organismal Level

After CAR-T cells are manufactured and intravenously infused, CAR-T cells proliferate and traffic between blood, lymph nodes, bone marrow, peripheral tissue, and tumor. *In vivo* detection and tracking of CAR-T cells can probe location-dependent phenotypes and elucidate models for therapy failure. There are three main methods for tracking CAR-T cells at the organismal level: bioluminescence imaging (BLI), positron emission tomography (PET) scanning, and two-photon microscopy (Figure 4B). All three methods can monitor CAR-T cells in organs. BLI and PET scanning utilize reporters and probes for visualization. A representative, but not exhaustive, list of applications of BLI and PET scanning is provided in Table 2. Two-photon microscopy utilizes fluorescence for visualization with single-cell resolution. Unlike CAR detection in the previous levels, CAR detection at the organismal level most directly studies CAR-T cells *in vivo*.

**Table 2:** Representative BLI and PET scanning applications.

**Method type**	**Reporter**	**Probe**	**Validation system**	**Notes and reference**
BLI	Firefly luciferase (FLuc)	D-luciferin	Anti-PSCA CAR-T cells in xenograft mouse model	(68)
BLI	Firefly luciferase (FLuc)	D-luciferin	anti-HLA-A*02:01 CAR-Tregs in human allograft mouse model	(69)
Duplex BLI	Renilla luciferase (RLuc) pavee Click beetle luciferase (CBRLuc)	Coelenterazine and D-luciferin	anti-PSMA CAR-T cells in xenograft mouse model	Signal diminished by poor substrate availability (70)
Duplex BLI	Stabilized color FLuc mutants	Infraluciferin	anti-CD19 CAR-T cells in xenograft mouse model	Used spectral unmixing (71)
PET scanning	Herpes simplex virus type 1 thymidine kinase (HSV1-TK)	^18^F-FHBG	IL-13 zetakine CAR-T cells in clinical trial	Clinical study (72)
PET Scanning	DOTA antibody reporter 1 (DAbR1)	^86^Y-AABD for imaging pavee ^177^Lu-AABD for suicide	anti-CD19 CAR-T cells in xenograft mouse model	Forms covalent bond between reporter and probe (73)
PET scanning	*E. coli* dihydrofolate reductase enzyme (eDHFR)	^18^F-labeled trimethoprim (^18^F-TMP)	anti-GD2 CAR-T cells in xenograft mouse model	Sensitivity of ~11,000 CD8^+^ CAR-T cells per mm^3^ (74)
PET Scanning	Human somatostatin receptor 2 (SSTR2)	^18^F-NOTA-Octreotide (NOTAOCT)	ICAM-1-directed CAR-T cells in xenograft mouse model	Background expression of SSTR2 in healthy human tissue (75, 76)
PET scanning	Human sodium iodide symporter (hNIS)	T99mcO4−	anti-PSMA CAR-T cells in xenograft mouse model	Cheap and widely used radiotracer (77)
PET scanning	Prostate-specific membrane antigen (PSMA)	^18^F-DCFPyL	anti-CD19 CAR-T cells in xenograft mouse model	Reporter/probe used extensively in tracking prostate cancer (78)
PET scanning	None	^89^Zr-*p*-isothiocyanatobenzyl-desferrioxamine (^89^Zr-DFO)	anti-CD19 CAR-T cells in xenograft mouse model	Physical labeling bypasses need for reporter; long half-life (79)

### Bioluminescence Imaging

*In vivo* BLI captures CAR-T cell biodistribution throughout an organism. Furthermore, BLI can probe for trafficking into solid tumors without needing to isolate the tumor for manual processing. To enable BLI, CAR-T cells must be co-transduced with luciferase. During imaging, luciferase substrate is injected, circulate and diffuse to CAR-T cells, and get processed by luciferase to emit light. The emitted light is captured using charge-coupled device (CCD) cameras, which convert light into electronic currents that localize the light source. Conventional luciferases come from terrestrial (i.e., North American firefly luciferase, FLuc) or marine (i.e., Renilla luciferase, RLuc) animals, which use D-luciferin or coelenterazine, respectively, along with O_2_ and sometimes ATP. Newer luciferases are smaller and more sensitive (80).

BLI can track *in vivo* CAR-T cell expansion. Torres Chavez et al. utilized BLI to compare expansion kinetics of anti-CD19 CAR-T cells cultured with different sera during *ex vivo* transduction. BLI showed that human platelet lysate led to memory-like CAR-T cells, which exhibited superior *in vivo* expansion upon tumor re-challenge (68). Furthermore, BLI can track *in vivo* CAR-T cell trafficking. Dawson et al. utilized BLI to show trafficking of anti-HLA-A^*^02:01 CAR-Tregs, which migrated into transplanted human allograft skin tissue and associated draining lymph nodes. Functionally, these CAR-Tregs prevented allograft rejection in NSG mice (69).

Importantly, luciferase/substrate pairs can be multiplexed. For example, FLuc and RLuc can tag different cell populations in the same animal for imaging using different substrates. Serganova et al. used multiplex BLI to simultaneously track anti-PSMA CAR-T and PSMA^+^ tumor cells with RLuc and click beetle luciferase, respectively, in a mouse model. Multiplex BLI revealed initial CAR-T cell sequestration in the lungs (70). However, this method requires sequential injection of luciferase substrates. The signal from the first injection must entirely disappear before the second injection, which requires careful optimization. Hence, Stowe et al. developed a different approach to BLI multiplexing: spectral unmixing. In their system, two cell populations are tagged with two distinct luciferases that share a common substrate: infraluciferin. These distinct luciferases generate light with dissimilar emission wavelengths. After infraluciferin injection, total BLI signal is captured, which is spectrally unmixed into two bioluminescence channels. Their method captured anti-CD19 CAR-T cells homing to and expanding within the lymphoma tumor in a mouse model (71). BLI spectral unmixing eliminates the requirement for sequential substrate injection.

Strengths of BLI include accessibility of CCD cameras among core facilities, standardized and high-throughput protocols, affordability, and multiplexed live-cell imaging. Furthermore, engineered FLuc derivatives, such as AkaLumine-HCl can emit near-infrared light, for superior deep-tissue penetration (81). Hence, BLI is the method of choice for preclinical CAR-T cell experiments. However, it comes with notable weaknesses. BLI is not used in clinical trials because humans are too large for the emitted light to penetrate tissue efficiently. Furthermore, the luciferase reporter may be immunogenic. In mice, unlike intravital two-photon microscopy, BLI cannot track CAR-T cells at the single-cell level. Finally, the location and metabolism of the CAR-T cells may decrease ATP and O_2_ availability, leading to diminished BLI signal. Substrate availability is even more limited in the tumor microenvironment (70). Engineered luciferases with superior enzymatic activity and tissue penetration only partially addresses these issues.

### Positron Emission Tomography

Positron emission tomography (PET) scanning also captures CAR-T cell biodistribution throughout an organism. To enable PET, the CAR is co-transduced with a PET reporter, which can capture and accumulate a positron-emitting small molecule probe. For imaging, the probe is intravenously injected, which preferentially accumulates in CAR-T cells due to the co-expressed PET reporter. Emitted positrons colocalize with CAR-T cells, lose kinetic energy, combine with a nearby electron, get annihilated, and emit high-energy photons. The high energy photons are captured with a PET scanner (82).

Although many PET reporter/probe pairs have been developed to track CAR-T cells, only one pair has been tried on patients in a CAR-T cell clinical study (NCT00730613 and NCT01082926): herpes simplex virus type 1 thymidine kinase (HSV1-TK) paired with 9-[4-[^18^F]fluoro-3-(hydroxymethyl)butyl]guanine (^18^F-FHBG). HSV1-TK is a cytosolic viral kinase that selectively phosphorylates nucleoside analogs such as ^18^F-FHBG. Phosphorylated ^18^F-FHBG then accumulates intracellularly. The pharmacology and safety profile of ^18^F-FHBG in humans are well-documented, and ^18^F-FHBG is FDA-approved as an investigational new drug. In this clinical study, Keu et al. co-expressed HSV1-TK with an interleukin-13 (IL-13) zetakine CAR in CD8^+^ T cells to treat recurrent high-grade glioma in seven patients. The glioma disrupts the blood-brain barrier, allowing ^18^F-FHBG to diffuse into the tumor. PET scans show increased signal around the tumor after CAR-T cell infusion, which suggests active trafficking of CAR-T cells into the tumor. However, increased PET signal can also feasibly be due to increased non-specific vascular leakage or glioma progression, which this pilot study cannot address (72). Importantly, the HSV1-TK reporter may also function as a suicide switch by accumulating ganciclovir, a separate nucleoside analog which can induce apoptosis (83). This can be a critical safety mechanism for patients experiencing adverse CAR-T cell-related side effects, including cytokine release syndrome and pneumonia. The kinetics and utility of HSV1-TK as a suicide switch for CAR-T cells have yet to be clinically tested.

Other PET reporter/probe pairs have been developed in preclinical mouse models and are summarized in Table 2. Krebs et al. (73) used DOTA antibody reporter 1 (DAbR1), which binds irreversibly on the cell surface with ^86^Y-labeled (*S*)-2-(4-acrylamidobenzyl)-DOTA (^86^Y-AABD). DAbR1 does not inhibit *in vitro* cytotoxicity, and PET scans show homing of CAR-T cells to the tumor. Furthermore, they predicted DAbR1 can be a suicide switch with ^177^Lu-AABD, a heavier and more radioactive nuclide. Sellmyer et al. used *Escherichia coli* dihydrofolate reductase enzyme (eDHFR), which binds to ^18^F-labeled trimethoprim (^18^F-TMP), to image anti-GD2 CAR-T cells in mouse xenograft models. PET scans show colocalization between anti-GD2 CAR-T cells and GD2^+^ tumor, which was confirmed with bioluminescence. Finally, they calculated that their method can detect ~11,000 CD8^+^ CAR-T cells per mm^3^ (74). Park et al. used human somatostatin receptor 2 (SSTR2), which ensures intracellular accumulation of ^18^F-NOTA-Octreotide (NOTAOCT), to track CAR-T cells of differing affinities to ICAM-1. PET scans captured CAR-T cell expansion and contraction kinetics (75). However, background expression of SSTR2 may preclude its use in clinical trials (76). Emami-Shahri et al. used human sodium iodide symporter (hNIS), which is compatible with T99mcO4−, a cheap and widely used clinical radiotracer. Their results show trafficking of anti-PSMA CAR-T cells into the tumor, which was confirmed by IHC (77). Finally, Minn et al. (78) co-transduced CAR-T cells with PSMA, which interacts with ^18^F-DCFPyL. Their results show divergence between CAR-T cell occupancy in blood, bone marrow, and tumor.

Furthermore, PET scanning can image physically labeled CAR-T cells, which bypasses the requirement for a PET reporter. CAR-T cells can be radiolabeled after manufacturing and prior to infusion. Lee et al. developed ^89^Zr-*p*-isothiocyanatobenzyl-desferrioxamine (Df-Bz-NCS, DFO), which covalently labels 70–79% of CAR-T cells with negligible impact on cell viability and proliferation. ^89^Zr's long half-life (78.4 h) makes this nuclide suitable for long-term *in vivo* tracking. PET scanning captured these cells as they migrated between lung, liver, and spleen (79).

Strengths of PET scanning include accessibility of PET scanners in the clinic, penetration of positrons through tissue, and the dual use of PET reporter also as a suicide safety switch. Unlike BLI, PET scanning is widely used in the clinic. However, PET scanning shares some limitations with BLI, including lack of single-cell resolution and potential immunogenicity of the PET reporter. The latter limitation can be ameliorated with PET reporters that are based on endogenous human proteins (e.g., SSTR2 and hNIS). However, background expression of endogenous human proteins may also obscure results. Finally, unlike with BLI, PET scanning cannot multiplex different reporters, since all reporter/probe pairs emit positrons. Hence, PET scanning cannot simultaneously image both CAR-T and tumor cells.

### Two-Photon Microscopy

Finally, two-photon microscopy can capture the distribution, motility, and functionality of CAR-T cells *in vivo* at the single-cell level. With two-photon microscopy, one fluorophore simultaneously absorbs multiple (usually two) units of near-infrared (NIR) photons and emits a single unit of fluorescence. Since NIR photons minimize scattering and multiphoton absorption occurs rarely in an area of high photon density, two-photon microscopy has deep tissue penetration, superior spatial resolution, and diminished photobleaching. These qualities are ideal for *in vivo* live-imaging mouse experiments to capture single CAR-T cells in action (84). Hence, out of the three CAR detection methods at the organismal level, two-photon microscopy is the most suitable for mechanistic studies at the cellular level.

Cazaux et al. utilized intravital two-photon microscopy to track GFP^+^CD8^+^ anti-murine CD19 CAR-T cells in a syngeneic lymphoma mouse model. In addition to CAR-T cell motility, two-photon microscopy was also used to measure calcium flux and detect apoptosis in CAR-T cells and cancer cells, respectively, via Förster resonance energy transfer sensors. Two-photon microscopy demonstrated that: (1) B cells in circulation hindered CAR-T cells from trafficking to the bone marrow; (2) CAR-T cells killed both directly (through contact) and indirectly (through epitope spreading or cytokines); and (3) there is less immunosurveillance in lymph nodes than in bone marrow (85). Furthermore, Mulazzani et al. used intravital two-photon microscopy to compare GFP^+^ anti-CD19 CAR-T cell infiltration into primary central nervous system lymphoma from intravenous and intracerebral CAR-T cell injection. Two-photon microscopy showed that intracerebral injection caused superior CAR-T cell infiltration and persistence, which was associated with long-term survival (86). In addition to genetically encoded fluorescent proteins, two-photon microscopy can also involve inorganic fluorophores. Ma et al. developed biodegradable polydopamine (PDA) nanodots with oxidation-induced fluorescence to track CAR-T cell targets *in vivo*. PDA can be endocytosed by target cells and oxidized intracellularly for imaging. They demonstrated proof-of-principle in dissected mouse tissue (87).

Two-photon microscopy is a powerful tool to capture *in vivo* CAR-T cell behavior and to generate novel hypotheses for CAR-T cell therapy failure and relapse. Its strengths (single-cell resolution, spatiotemporal resolution, tissue penetration) are ideal for mechanistic studies in mice. In addition, this method naturally links with other fluorescence-based tools, such as FRET sensors. However, unlike PET-based CAR tracking, two-photon microscopy cannot realistically be applied for clinical studies. Furthermore, two-photon microscopy requires equipment that might be inaccessible for many labs.

## Discussion and Outlook

In this review, we summarized CAR detection methods that operate at the genomic, transcriptomic, proteomic, and organismal levels. We have also identified key areas where CAR detection methods may be improved.

Based on the studies summarized in this review, we observed that development of new CAR detection methods has often proceeded through three phases. First, the new detection method is tested, validated, and optimized using CAR-T cells generated from a healthy donor's T cells after transduction with a known CAR construct. Under this controlled scenario, the method's accuracy (e.g., false negative and false positive incidences) and reproducibility (e.g., error across replicates) can be measured and optimized. Second, the optimized detection method is applied on clinical CAR-T cell samples from patients. Successful application on clinical samples demonstrates utility in a real-world scenario. Third, the results from the new detection method are compared with results obtained from existing detection methods. These three phases measure performance metrics and ensure utility for clinical studies.

Although no standard guidelines exist for developing new CAR detection methods, we believe the three aforementioned phases can serve as practical guidelines for development of future CAR detection methods. Furthermore, if the detection method is to be used for clinical or diagnostic purposes, we believe it should be accurate, reliable, reproducible, readily implemented, and easily interpreted. Results from one clinical laboratory should be replicable in another clinical laboratory. These suggestions are complementary with GCLP practices.

For CAR basic science studies, we believe that developing single-molecule microscopy-based CAR visualization will become increasingly important. The CAR has existed in its current form for years, but its molecular mechanisms are poorly understood or optimized. Although functionally similar to the TCR, the CAR traffics differently (62), and signaling is less efficient and sensitive (88). Furthermore, CAR-induced tonic signaling hastens CAR-T cell exhaustion (24, 38). We believe CAR signaling inefficiencies should be understood via microscopy-based CAR IS visualization. Mechanistic insights from CAR IS visualization with new technologies, such as super-resolution or lattice light-sheet microscopy, may inform engineering endeavors that improve CARs.

For CAR clinical studies, we believe that developing PET-based *in vivo* CAR-tracking methods will become increasingly important. Since multiple clinical trials aim to extend CAR-T cell therapy from hematological cancers to solid tumors, the ability to measure CAR-T cell trafficking into the tumor without the need for a biopsy is essential. This is particularly important for tumors at physically hard-to-access locations. The recent clinical trial that utilizes HSV1-TK as a PET reporter is a promising start, but confounding variables (vascular leakage and glioma progression) obscured conclusions drawn from their data (72). Newer CAR-T cell clinical studies that involve solid tumors should routinely employ PET scanning as both a research tool and on-treatment indicator of clinical efficacy. Meanwhile, other clinical studies should address the potential use of PET reporters as a CAR suicide safety switch.

## Author Contributions

JH conceived the original concept. YH organized and wrote the manuscript. All authors contributed to the article and approved the submitted version.

## Conflict of Interest

The authors declare that the research was conducted in the absence of any commercial or financial relationships that could be construed as a potential conflict of interest.
